# Supplementary material for: Multivariate discovery and replication of five novel loci associated with Immunoglobulin G N-glycosylation
Source: Nat Commun. 2017 Sep 6;8:447. doi: 10.1038/s41467-017-00453-3 (PMC5587582; doi:10.1038/s41467-017-00453-3)
Supplement: Supplementary file 2 — Supplementary Information [file 41467_2017_453_MOESM2_ESM.pdf]

Description of Supplementary Files

File name: Supplementary Information
Description: Supplementary figures, supplementary tables, supplementary note and supplementary references.

File name: Supplementary Data 1
Description: Detailed multivariate analysis results of the novel loci in discovery and replication cohorts.

File name: Supplementary Data 2
Description: DEPICT results for the identified loci associated with IgG *N*-glycosylation.

File name: Peer review file

Supplementary Note 1: Description of multivariate methods

Many multivariate methods have been developed for genetic analyses (reviewed by [1]) – specifically, for GWAS, there are two main approaches (**Supplementary Table 1**; see also an earlier review by [2]). The first is *P*-value adjustment for univariate GWAS results, such as Pact [3] and TATES [4], which do not directly incorporate the genetically-regulated phenotypic covariance in the model. The second type of method models relations between multiple phenotypes and the genotype simultaneously; examples of these are MQFAM [5] in PLINK [6] (plink.multivariate with options --mult-pheno and --mqfam), MultiPhen [7] and --mpheno option in SNPTEST [8]. The first methodology was developed for efficient analysis of GWAS data, however, the latter approach is preferable since a full likelihood containing multiple traits and the tested SNP is considered. In fact, MultiPhen is mathematically equivalent to a multivariate analysis of variance (MANOVA) for a single genetic variant, and MQFAM is equivalent to a MANOVA with Wilk's lambda as the test statistic (**Supplementary Fig. 8**). Application of MANOVA has a long tradition in the field of quantitative genetics, e.g. more than a decade ago, it was proposed for quantitative trait loci (QTL) mapping [9]. The MANOVA-based methods described above can handle population stratification by including principle components of the genomic kinship matrix in the model. However, the effects of small groups of close (even first-degree) relatives are usually not reflected in the leading principal components because the (cryptic) kinship generates weaker LD than large-scale population structure [10]. In such situations, the method of choice is mixed effect models; however, a fast mixed model analysis for multiple phenotypes is still difficult. GEMMA [11] and Limix [12] were designed to overcome such difficulty, though the computational cost can be high for a large number of phenotypes such as that encountered in omics data. Another difficulty faced in multivariate analysis of omics phenotypes is finding a method for replication, which would allow for both tests of significance and consistency of the model

being replicated.

The implementation here (i) transforms multiple phenotypes prior to multivariate analysis to correct for population stratification, so that a larger number of phenotypes can be analyzed simultaneously; (ii) estimates joint genetic effects of the phenotypes, i.e. the partial correlations between the genotype and multiple phenotypes, so that the replication can be performed more meaningfully, and the pleiotropic model consistency can be examined.

Supplementary References

1. Shriner, D. Moving toward System Genetics through Multiple Trait Analysis in Genome-Wide Association Studies. *Front Genet* **3,** 1 (2012).
2. Galesloot, T. E., van Steen, K., Kiemeney, L. A. L. M., Janss, L. L. & Vermeulen, S. H. A Comparison of Multivariate Genome-Wide Association Methods. *PLoS ONE* **9,** e95923 (2014).
3. Conneely, K. N. & Boehnke, M. So many correlated tests, so little time! Rapid adjustment of P values for multiple correlated tests. *Am. J. Hum. Genet.* **81,** 1158–1168 (2007).
4. van der Sluis, S., Posthuma, D. & Dolan, C. V. TATES: efficient multivariate genotype-phenotype analysis for genome-wide association studies. *PLoS Genet.* **9,** e1003235 (2013).
5. Ferreira, M. & Purcell, S. M. A multivariate test of association. *Bioinformatics* (2009).
6. Purcell, S. *et al.* PLINK: a tool set for whole-genome association and population-based linkage analyses. *Am. J. Hum. Genet.* **81,** 559–575 (2007).
7. O'Reilly, P. F. *et al.* MultiPhen: joint model of multiple phenotypes can increase discovery in GWAS. *PLoS ONE* **7,** e34861 (2012).
8. Marchini, J., Howie, B., Myers, S., McVean, G. & Donnelly, P. A new multipoint method for genome-wide association studies by imputation of genotypes. *Nature Genetics* **39,** 906–913 (2007).
9. Knott, S. A. & Haley, C. S. Multitrait least squares for quantitative trait loci detection. *Genetics* **156,** 899–911 (2000).
10. Astle, W. & Balding, D. J. Population structure and cryptic relatedness in genetic association studies. *Statistical Science* **24,** 451–471 (2009).
11. Zhou, X. & Stephens, M. Efficient multivariate linear mixed model algorithms for genome-wide association studies. *Nat. Methods* **11,** 407–409 (2014).
12. Casale, F. P., Rakitsch, B., Lippert, C. & Stegle, O. Efficient set tests for the genetic analysis of correlated traits. *Nat. Methods* **12,** 755–758 (2015).

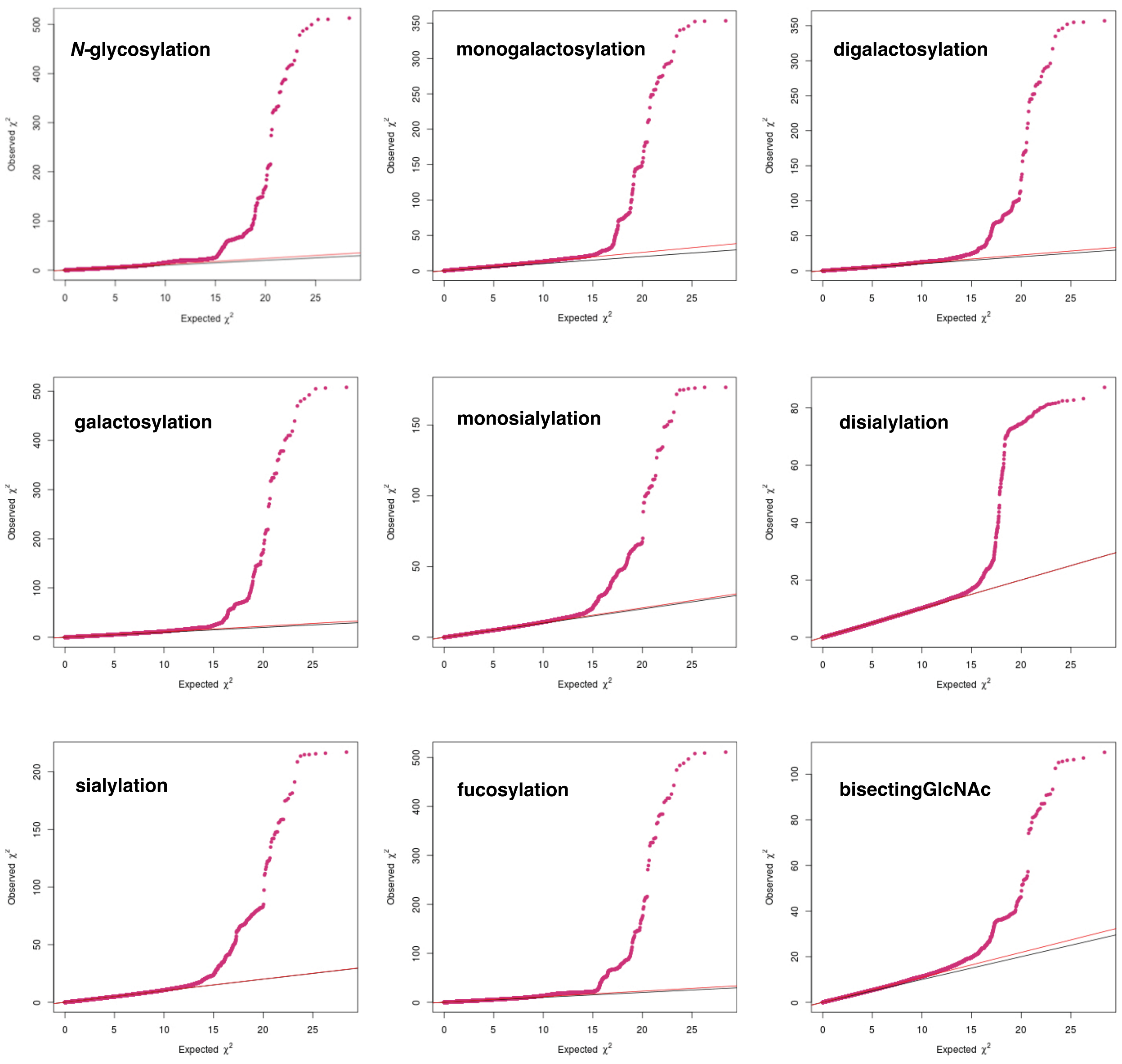

Supplementary Figure 1: The Quantile-Quantile plots of the discovery GWA analyses results. The regression line of the observed against the theoretical chi-squared is shown in red. The black line indicates equality.

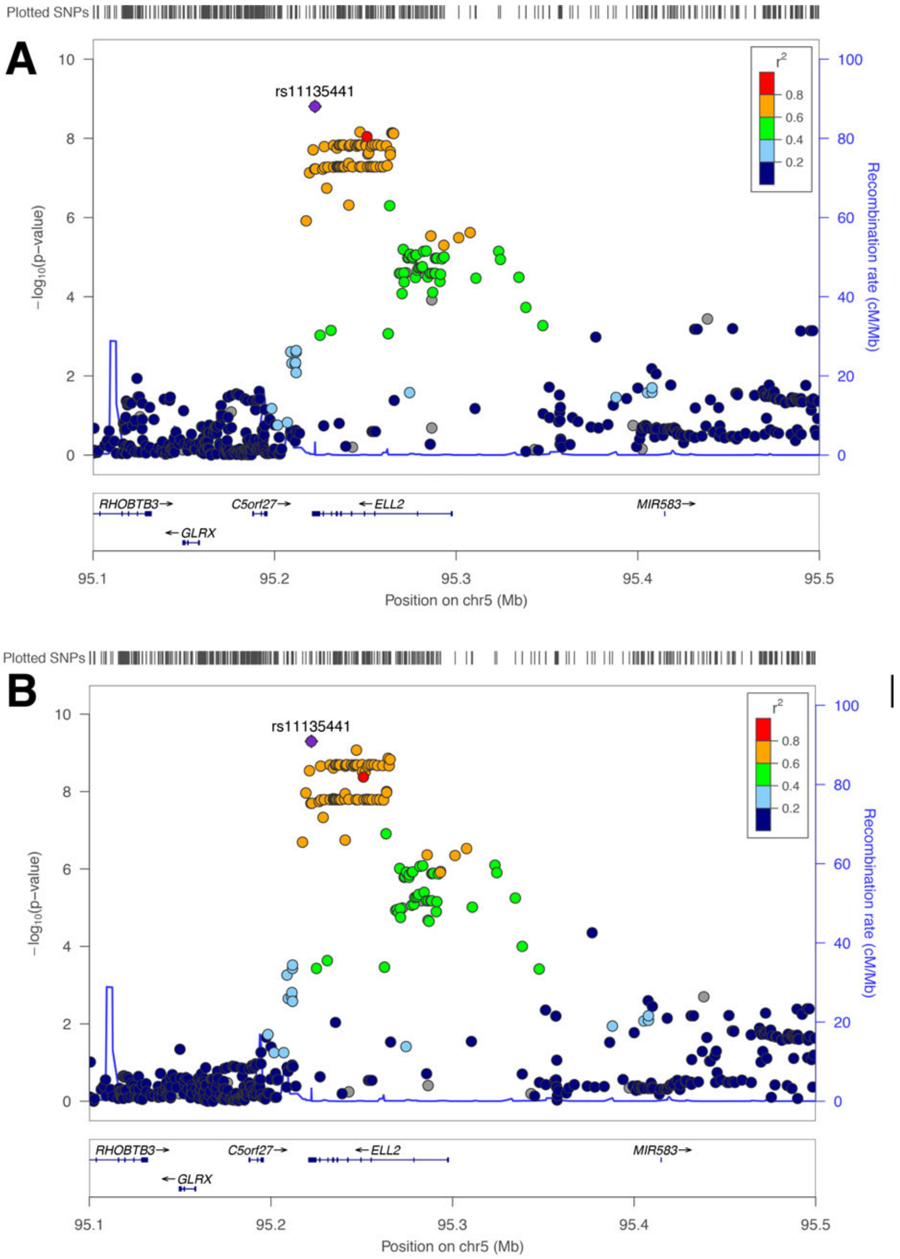

Supplementary Figure 2: The regional association pattern at locus *ELL2*. (A) Digalactosylation phenotypes; (B) Sialylation phenotypes.

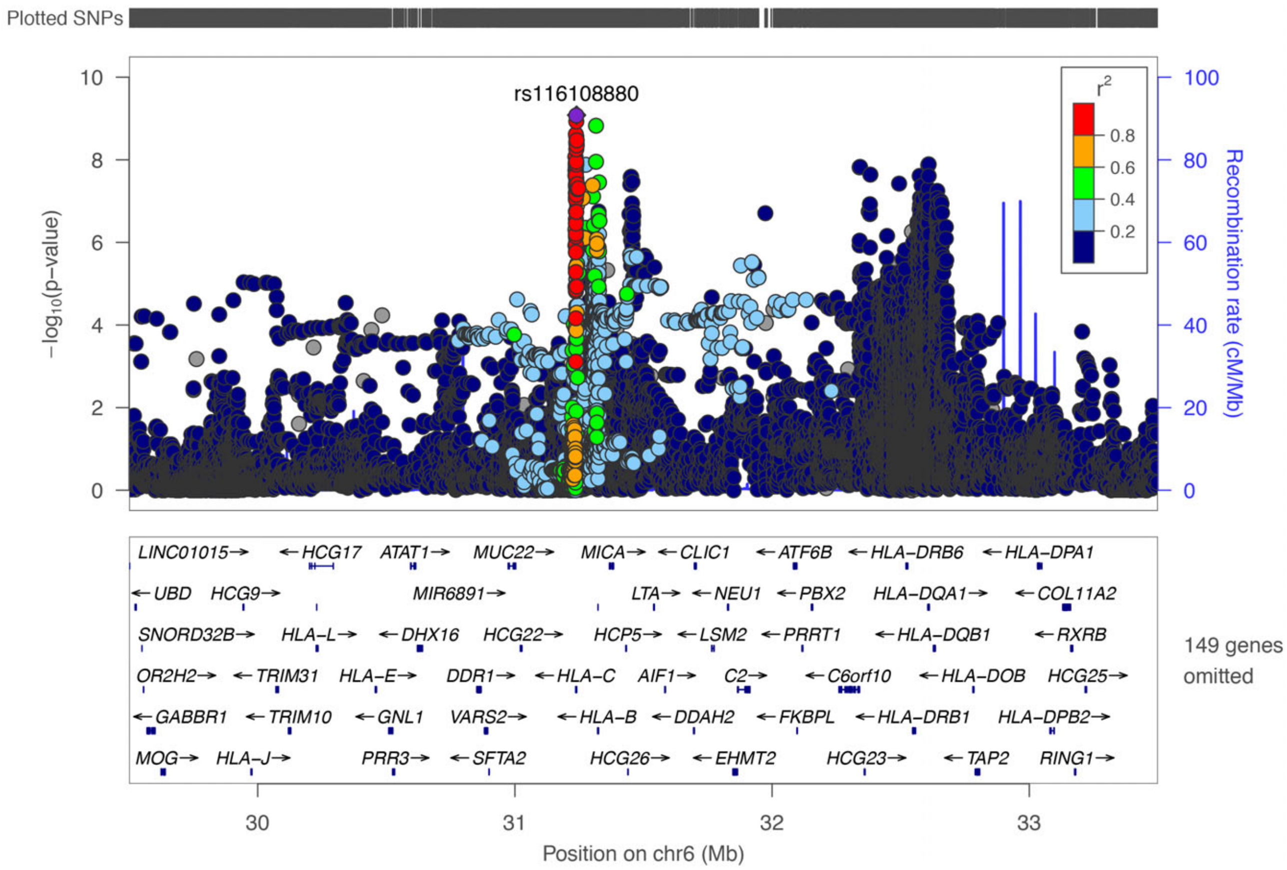

Supplementary Figure 3: The regional association pattern at locus *HLA-B, HLA-C* for the IgG galactosylation phenotypes.

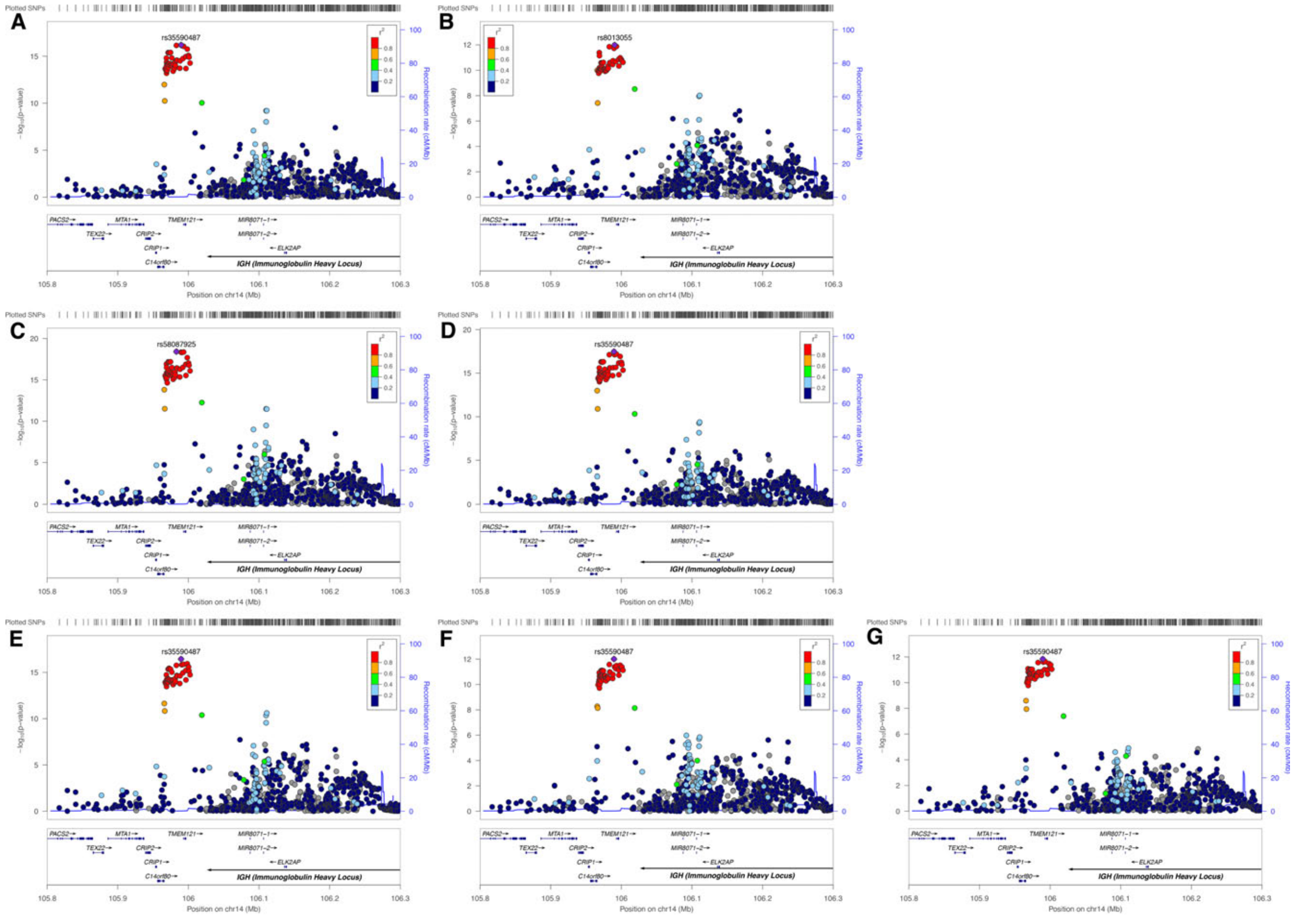

Supplementary Figure 4: The regional association pattern at locus *IGH*. (A) All the *N*-glycosylation phenotypes; (B) Bisecting GlcNAc phenotypes; (C) Fucosylation phenotypes; (D) Galactosylation phenotypes; (E) Monogalactosylation phenotypes; (F) Monosialylation phenotypes; (G) Sialylation phenotypes.

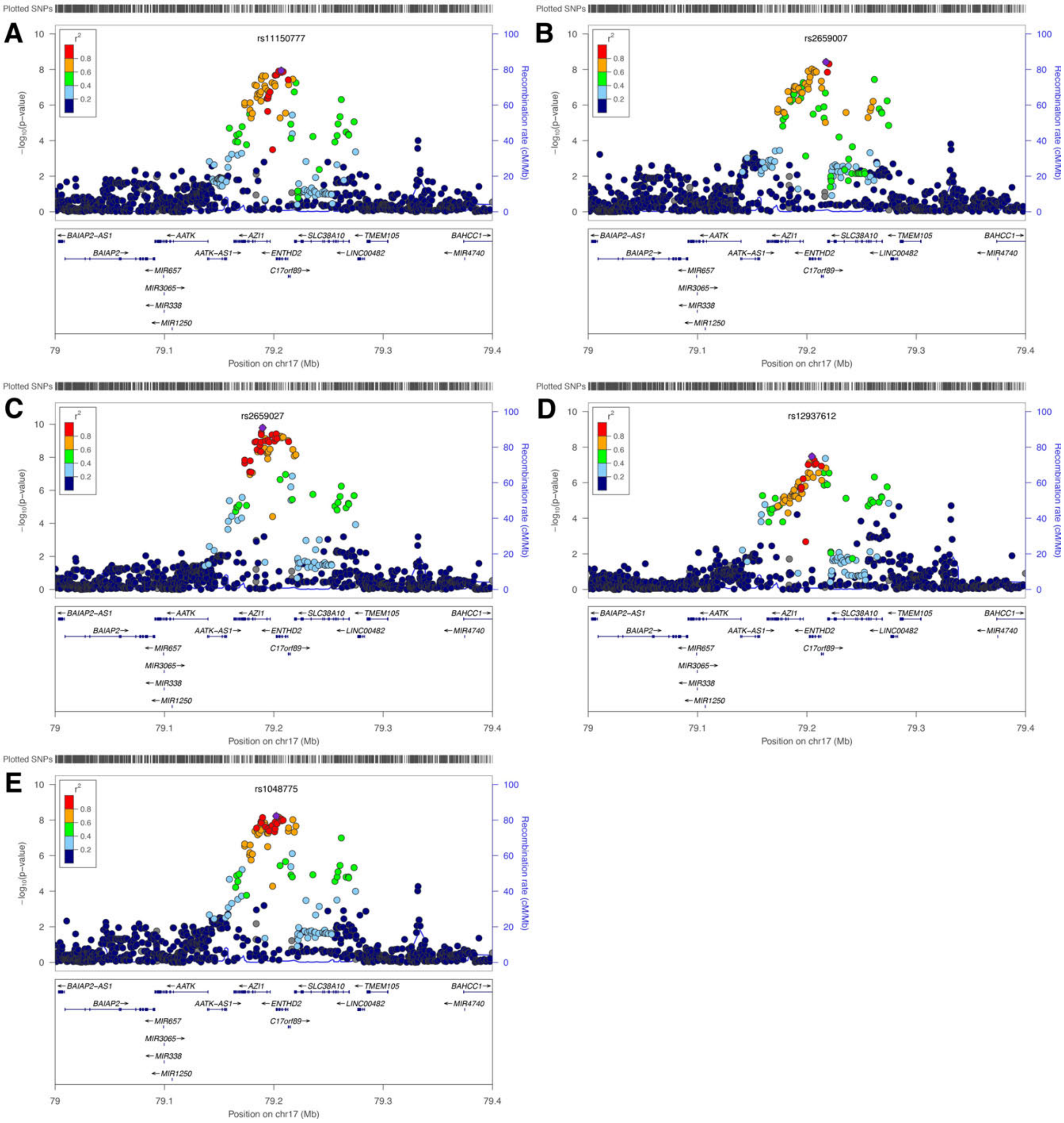

Supplementary Figure 5: The regional association pattern at locus *AZI1*. (A) All the *N*-glycosylation phenotypes; (B) Digalactosylation phenotypes; (C) Fucosylation phenotypes; (F) Monogalactosylation phenotypes; (G) Galactosylation phenotypes.

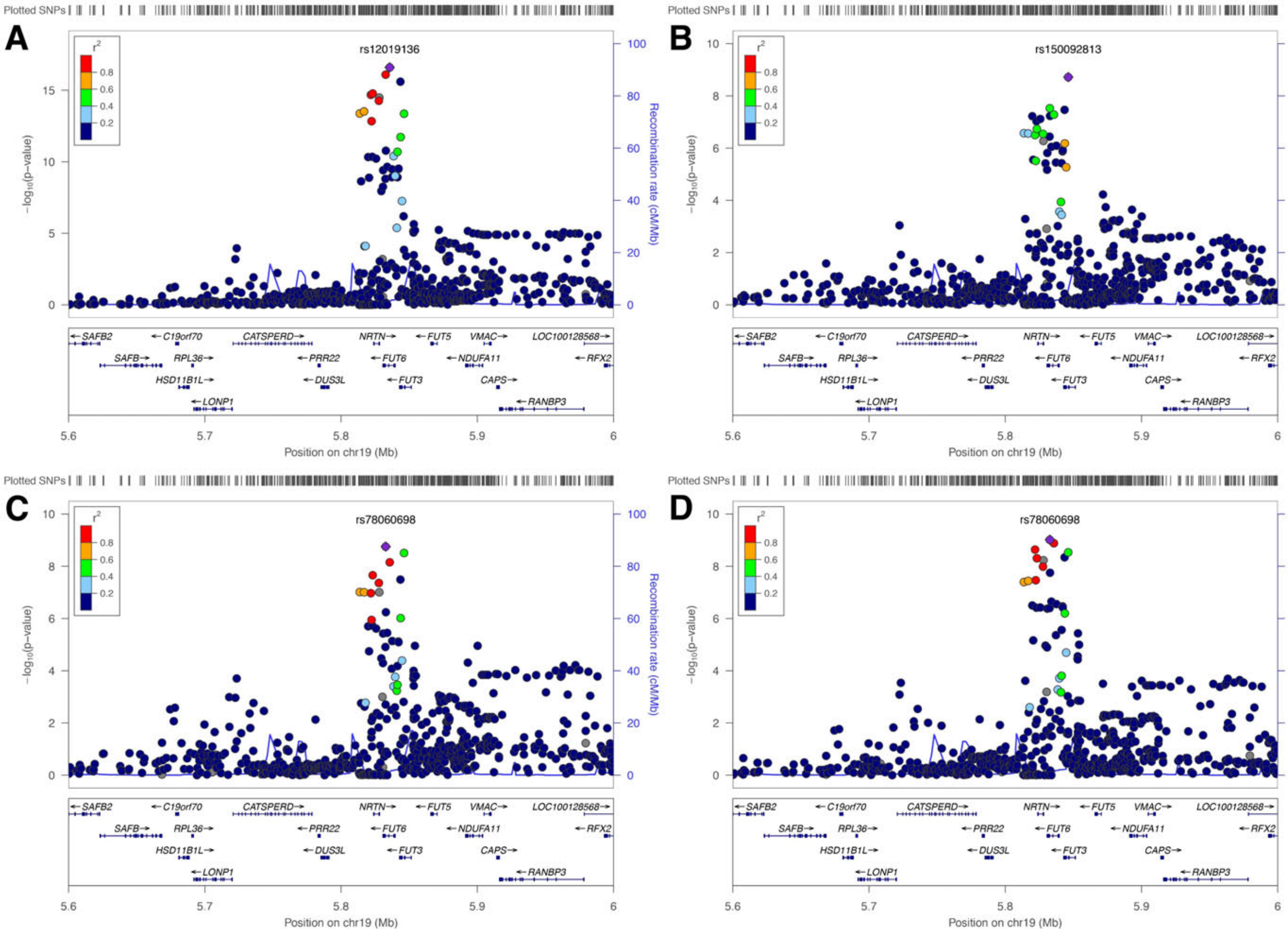

Supplementary Figure 6: The regional association pattern at locus *FUT6*. (A) All the *N*-glycosylation phenotypes; (B) Bisecting GlcNAc phenotypes; (C) Digalactosylation phenotypes; (D) Galactosylation phenotypes.

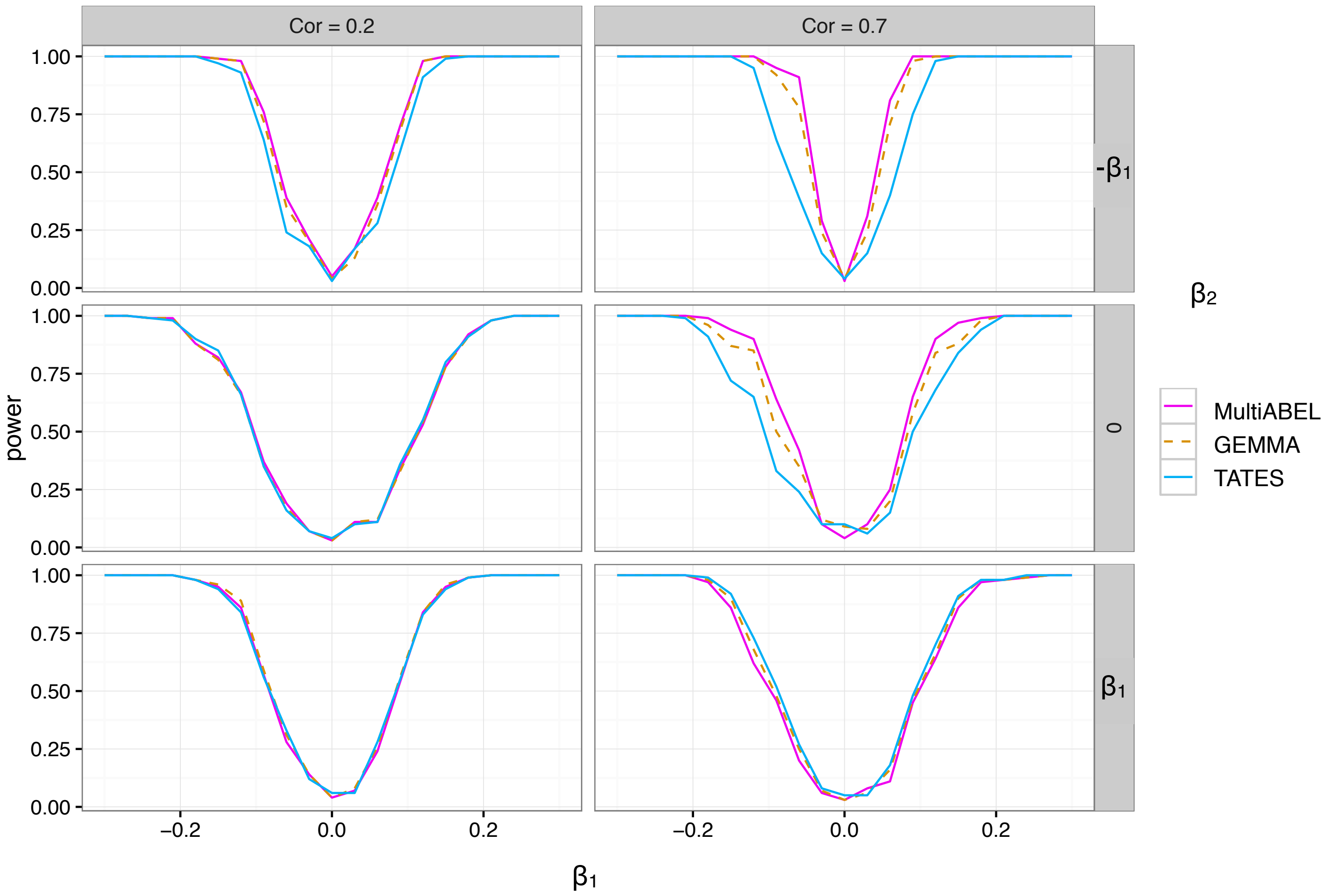

Supplementary Figure 7: Bivariate simulation comparing the statistical power of MultiABEL (GRAMMAR+ and MANOVA), GEMMA and TATES for unrelated individuals. β: univariate genetic effects; Cor: phenotypic correlation. Each point was generated by 100 replicates of the simulated data, $n = 2\,000$.

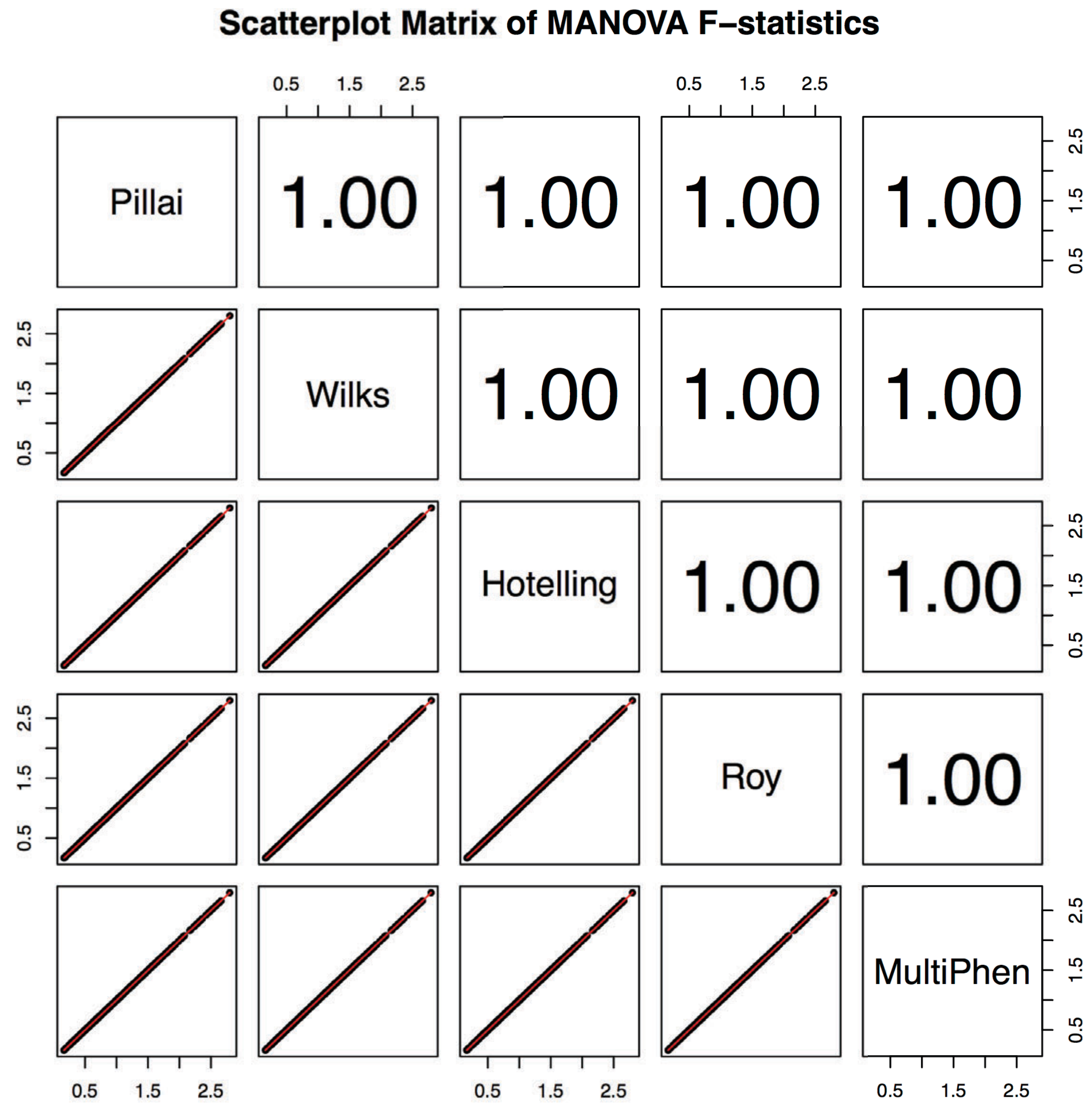

Supplementary Figure 8: Comparison of the corresponding F-statistics of four different MANOVA test statistics and the multiple regression F-statistic used by MultiPhen. 2000 individuals were simulated with 10 correlated phenotypes and a single SNP genotype (MAF = 0.3). Different ways of calculating the F-statistics were used, and the simulation was replicated for 1000 times to generate the scatterplot, and the upper triangle gives the correlation coefficients estimates across 1000 realizations.

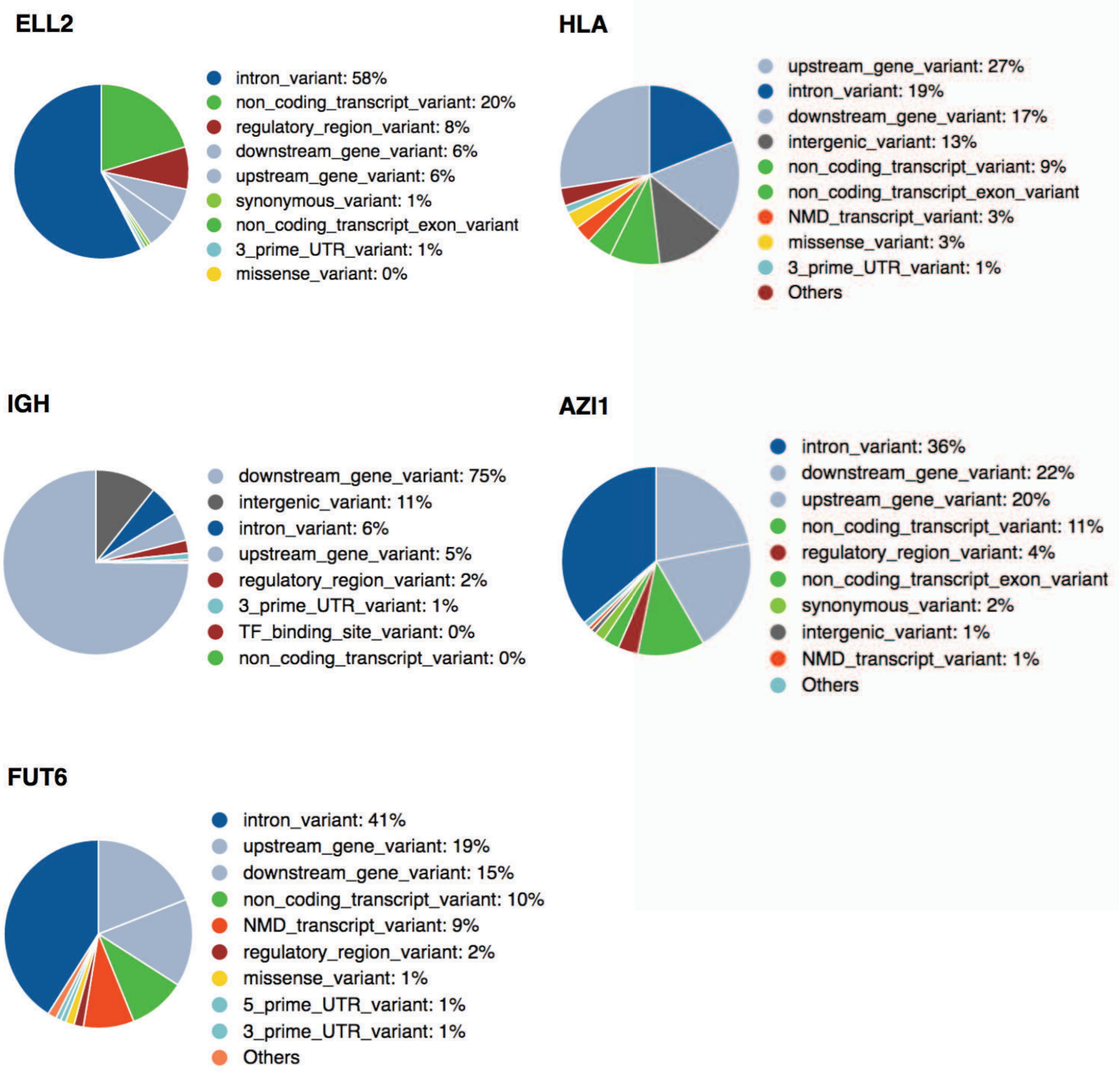

Supplementary Figure 9: Ensemble variants effects prediction summary for the five novel genome-wide significant loci.

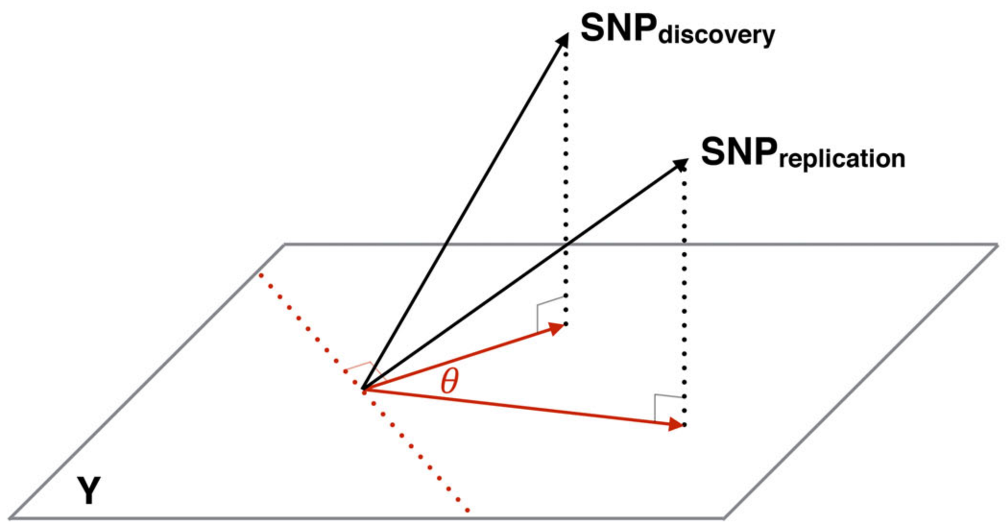

Supplementary Figure 10: Geometrical illustration comparing genotype-phenotypes relationship in discovery and replication samples. Y: a group of phenotypic vectors.

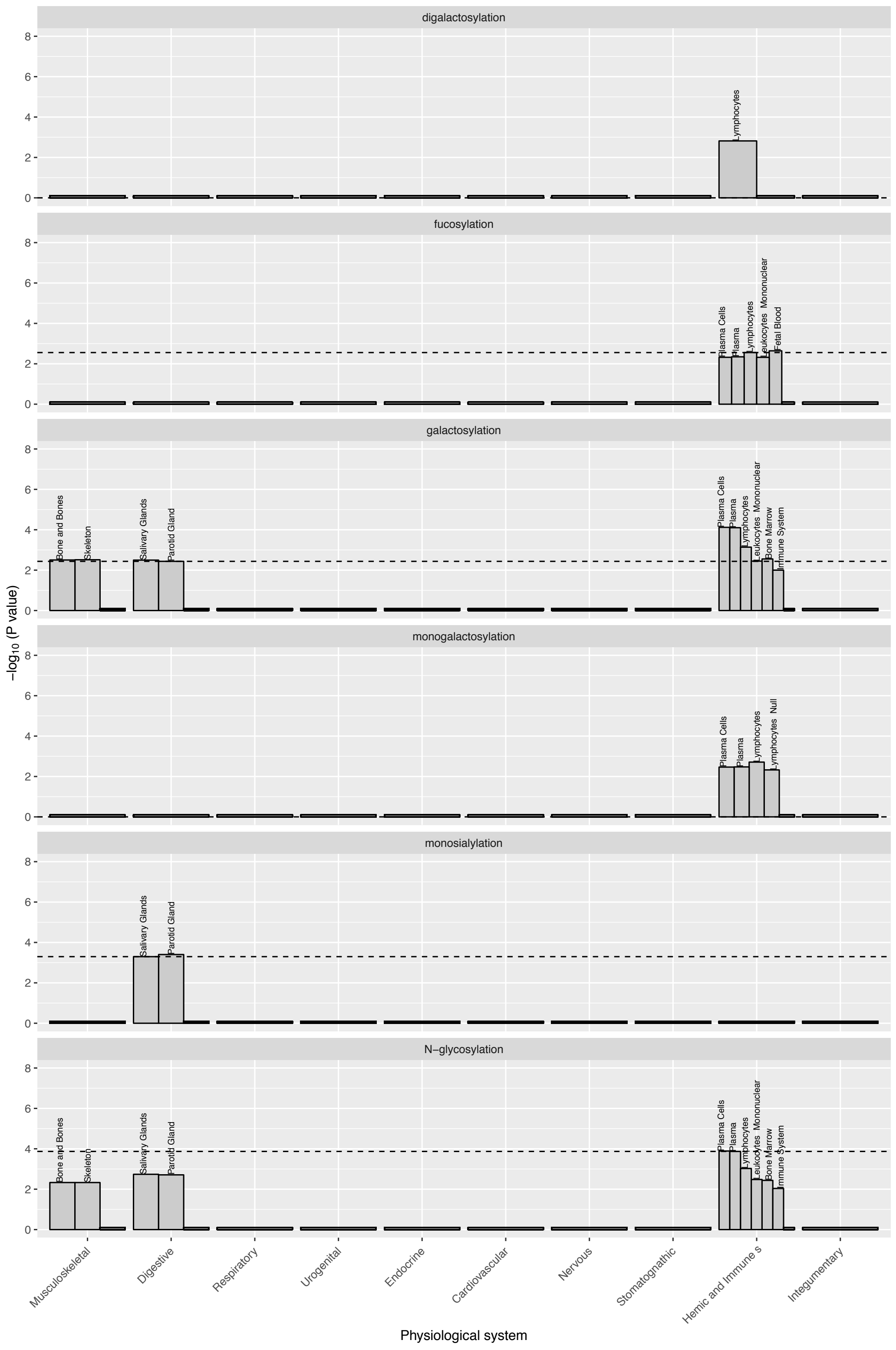

Supplementary Figure 11: IgG glycan group-specific enrichment of physiological systems using DEPICT.

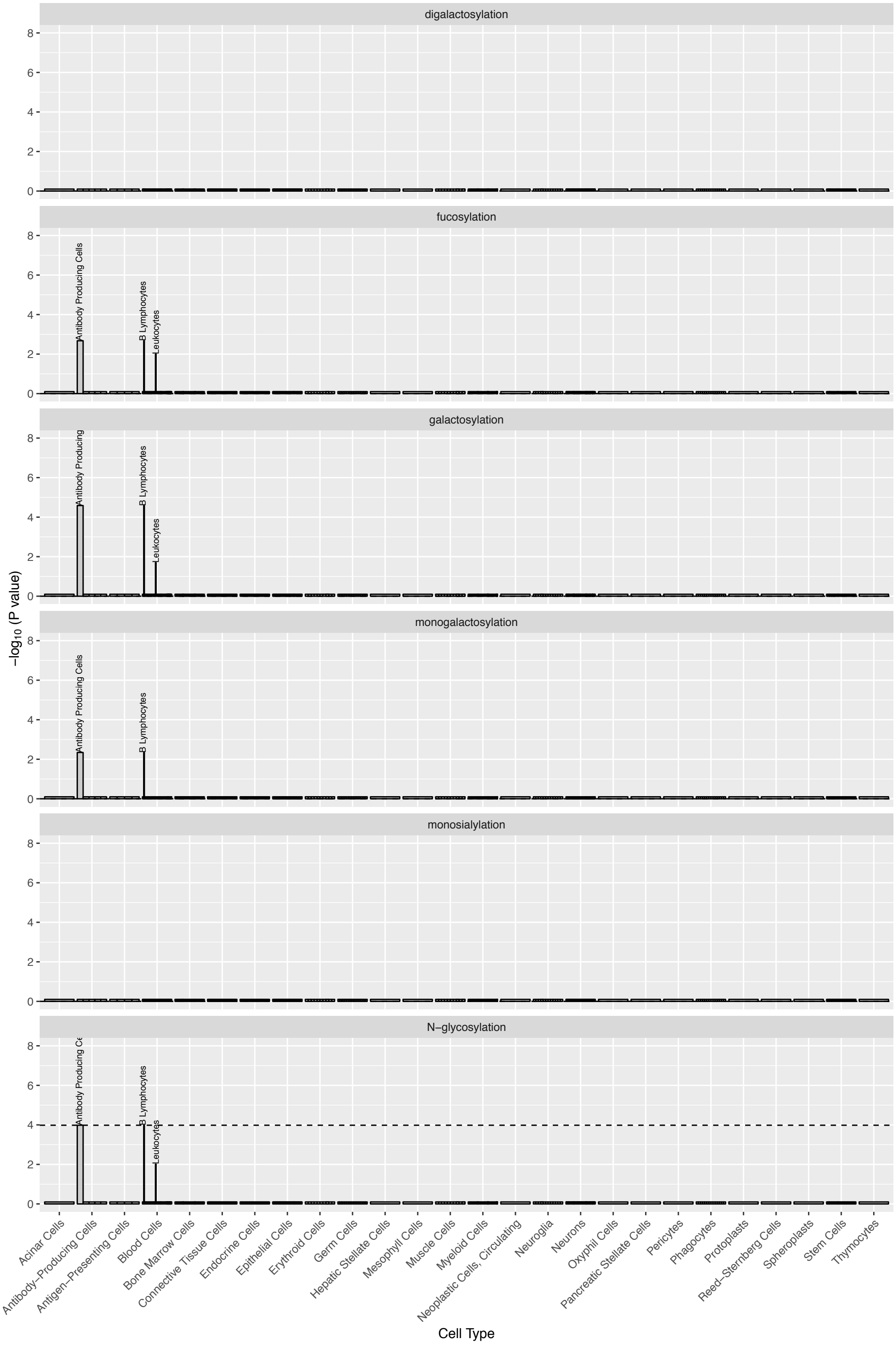

Supplementary Figure 12: IgG glycan group-specific enrichment of cell types using DEPICT.

Supplementary Table 1: Comparison of a set of different multi-trait GWAS methods.

Method	Joint Genetic Effect	Joint *P*-value	Conditional Genetic Effect per Trait	Conditional *P*-value per Trait	Best Linear Combination of Traits	Multivariate Test	Pros	Cons
**PLINK**: multivariate.plink	No	Yes	No	No	No	MANOVA	No specific	Population structure can only be handled via principle components as covariates.
**MultiPhen**	No	Yes	No	No	Yes	Multiple regression F-test	No specific	Population structure can only be handled via principle components as covariates.
**SNPTEST**: mpheno	No	Yes	No	No	No	Bayesian multiple phenotype test	No specific	Population structure can only be handled via principle components as covariates.
**TATES**	No	Yes	No	No	No	Adjustment of single-trait p-values	Only single-trait p-values and phenotypic correlations needed.	No genetic effects analyzed. Sacrifices discovery power.
**GEMMA**	No	Yes	Yes*	Yes*	No	Multivariate linear mixed models	Powerful 1-step mixed modeling to handle population structure.	Computationally heavy when the number of phenotypes is > 10.
**Limix**: mtSet	No	Yes	No	No	No	Set test	Fast implementation for a large number of individuals.	Numerical optimization algorithm may produce unstable estimates.
**MultiABEL**: Multivariate	Yes	Yes	Yes	Yes	Yes	Mixed model GRAMMAR+ residuals & MANOVA	Mixed model GRAMMAR+ residuals to handle population structure for a big number of phenotypes. Compatible with *ABEL data formats.	May lose relatively more power compared to GEMMA when the degree of relatedness is very high in the population.

Supplementary Table 2: Definitions and brief descriptions of nine groupings of IgG glycosylation phenotypes.

TRAIT	GLYCAN STRUCTURE	DESCRIPTION	FORMULA
GP1	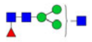	*The percentage of FA1 glycan in total IgG glycans*	*GP1 / GP * 100*
GP2	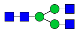	*The percentage of A2 glycan in total IgG glycans*	*GP2 / GP * 100*
GP4	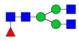	*The percentage of FA2 glycan in total IgG glycans*	*GP4 / GP * 100*
GP5	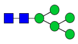	*The percentage of M5 glycan in total IgG glycans*	*GP5 / GP * 100*
GP6	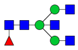	*The percentage of FA2B glycan in total IgG glycans*	*GP6 / GP * 100*
GP7	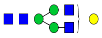	*The percentage of A2G1 glycan in total IgG glycans*	*GP7 / GP * 100*
GP8	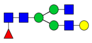	*The percentage of FA2[6]G1 glycan in total IgG glycans*	*GP8 / GP * 100*
GP9	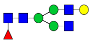	*The percentage of FA2[3]G1 glycan in total IgG glycans*	*GP9 / GP * 100*
GP10	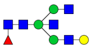	*The percentage of FA2[6]BG1 glycan in total IgG glycans*	*GP10 / GP * 100*
GP11	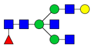	*The percentage of FA2[3]BG1 glycan in total IgG glycans*	*GP11 / GP * 100*
GP12	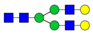	*The percentage of A2G2 glycan in total IgG glycans*	*GP12 / GP * 100*
GP13	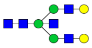	*The percentage of A2BG2 glycan in total IgG glycans*	*GP13 / GP * 100*
GP14	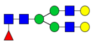	*The percentage of FA2G2 glycan in total IgG glycans*	*GP14 / GP * 100*
GP15	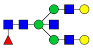	*The percentage of FA2BG2 glycan in total IgG glycans*	*GP15 / GP * 100*
GP16	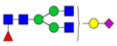	*The percentage of FA2G1S1 glycan in total IgG glycans*	*GP16 / GP * 100*
GP17	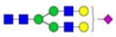	*The percentage of A2G2S1 glycan in total IgG glycans*	*GP17/ GP * 100*
GP18	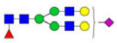	*The percentage of FA2G2S1 glycan in total IgG glycans*	*GP18 / GP * 100*
GP19	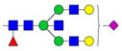	*The percentage of FA2BG2S1 glycan in total IgG glycans*	*GP19 / GP * 100*
GP20	Structure not determined	*Structure not determined*	*GP20 / GP * 100*
GP21	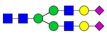	*The percentage of A2G2S2 glycan in total IgG glycans*	*GP21 / GP * 100*
GP22	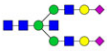	*The percentage of A2BG2S2 glycan in total IgG glycans*	*GP22 / GP * 100*
GP23	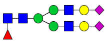	*The percentage of FA2G2S2 glycan in total IgG glycans*	*GP23 / GP * 100*
GP24	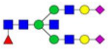	*The percentage of FA2BG2S2 glycan in total IgG glycans*	*GP24 / GP * 100*

Groups	**N-glycosylation**	GP1, GP2, GP4, GP5, GP6, GP7, GP8, GP9, GP10, GP11, GP12, GP13, GP14, GP15, GP16, GP17, GP18, GP19, GP20, GP21, GP22, GP23, GP24
	**monogalactosylation**	GP7, GP8, GP9, GP10, GP11, GP16
	**digalactosylation**	GP12, GP13, GP14, GP15, GP17, GP18, GP19, GP21, GP22, GP23, GP24
	**galactosylation**	GP7, GP8, GP9, GP10, GP11, GP12, GP13, GP14, GP15, GP16, GP17, GP18, GP19, GP21, GP22, GP23, GP24
	**monosialylation**	GP16, GP17, GP18, GP19
	**disialylation**	GP21, GP22, GP23, GP24
	**sialylation**	GP16, GP17, GP18, GP19, GP21, GP22, GP23, GP24
	**fucosylation**	GP1,GP4, GP6, GP8, GP9, GP10, GP11, GP14, GP15,GP16, GP18, GP19, GP23, GP24
	**bisectingGlcNAc**	GP6, GP10, GP11, GP13, GP15, GP19, GP24

Supplementary Table 3: Detected known loci via multivariate GWAS for IgG N-glycosylation phenotypes. Nine multivariate GWA scans were performed, including one using all the 23 phenotypes (N-glycosylation) and the other eight different groupings according to glycosylation types. The results, in terms of discovery P-values (Pmulti), were compared to P-value adjustment method TATES (PTATES) and univariate GWAS (Puni). f(): frequency; R2: imputation R-squared.

Chromosome	Reported Variant	Candidate Genes	A1	A0	f(A1)	R^2	Phenotype Grouping	Cohort: ORCADES		
								min(P_{uni})	P_{TATES}	P_{multi}
Genome-wide Significant in Multivariate GWAS										
3	rs11710456	*ST6GAL1*	A	G	0.29	0.99	*N*-glycosylation	1.74E-42	3.20E-41	5.73E-87
							monogalactosylation	1.74E-42	9.72E-42	2.52E-69
							digalactosylation	6.66E-10	6.00E-09	5.49E-79
							galactosylation	1.74E-42	2.46E-41	1.79E-90
							monosialylation	1.74E-42	6.79E-42	6.59E-42
							disialylation	3.38E-09	1.16E-08	2.39E-13
							sialylation	1.74E-42	1.22E-41	2.06E-51
							fucosylation	1.74E-42	2.00E-41	1.21E-88
							bisecting GlcNAc	3.38E-09	2.04E-08	2.00E-24
9	rs12342831	*B4GALT1*	C	T	0.29	1.00	*N*-glycosylation	9.55E-06	1.76E-04	1.41E-10
							monogalactosylation	5.23E-02	2.84E-01	1.12E-01
							digalactosylation	9.55E-06	8.60E-05	1.22E-03
							galactosylation	9.55E-06	1.35E-04	7.02E-08
							monosialylation	9.55E-06	3.74E-05	6.14E-05
							disialylation	2.21E-03	7.59E-03	1.11E-02
							sialylation	9.55E-06	7.73E-05	3.99E-04
							fucosylation	9.55E-06	1.10E-04	5.02E-13
							bisecting GlcNAc	1.85E-01	5.98E-01	1.36E-01
14	rs11847263	*FUT8*	G	T	0.35	0.99	*N*-glycosylation	8.19E-14	1.51E-12	2.99E-15
							monogalactosylation	3.19E-13	1.79E-12	1.18E-17
							digalactosylation	8.19E-14	7.38E-13	3.27E-16
							galactosylation	8.19E-14	1.16E-12	1.11E-15
							monosialylation	8.19E-14	3.21E-13	5.89E-15
							disialylation	2.90E-02	9.97E-02	1.13E-05
							sialylation	8.19E-14	5.78E-13	5.40E-15
							fucosylation	2.16E-03	2.50E-02	5.90E-10
							bisecting GlcNAc	3.73E-02	1.82E-01	2.05E-04
22	rs2186369	*SMARCB1*<br>*DERL3*	G	T	0.13	0.96	*N*-glycosylation	8.21E-11	1.52E-09	3.03E-11
							monogalactosylation	8.21E-11	4.60E-10	1.45E-12
							digalactosylation	7.81E-06	7.03E-05	4.13E-10
							galactosylation	8.21E-11	1.16E-09	2.66E-10
							monosialylation	6.64E-02	2.35E-01	2.70E-01
							disialylation	4.59E-03	1.58E-02	1.07E-07
							sialylation	4.58E-03	3.23E-02	1.06E-06
							fucosylation	8.21E-11	9.48E-10	3.36E-11
							bisecting GlcNAc	8.21E-11	4.95E-10	3.86E-11
22	rs909674	*SYNGR1*<br>*TAB1*<br>*MGAT3*	A	C	0.78	0.92	*N*-glycosylation	3.17E-10	5.85E-09	2.92E-19
							monogalactosylation	2.33E-08	1.30E-07	1.39E-06
							digalactosylation	3.17E-10	2.85E-09	3.12E-13
							galactosylation	3.17E-10	4.48E-09	2.45E-14
							monosialylation	1.19E-01	4.64E-01	5.41E-01
							disialylation	3.17E-10	1.09E-09	3.85E-14
							sialylation	3.17E-10	2.23E-09	1.34E-13
							fucosylation	3.17E-10	3.66E-09	1.13E-20
							bisecting GlcNAc	2.33E-08	1.40E-07	3.83E-10
Suggestively Significant in Multivariate GWAS										
7	rs6421315	*IKZF1*	C	G	0.39	0.86	*N*-glycosylation	4.10E-04	7.57E-03	1.65E-07
							monogalactosylation	6.68E-03	1.86E-02	3.44E-06
							digalactosylation	4.10E-04	3.69E-03	1.65E-07
							galactosylation	4.10E-04	5.80E.03	1.32E-08
							monosialylation	6.68E-03	2.61E-02	2.95E-04
							disialylation	4.10E-04	1.41E-03	2.52E-07
							sialylation	4.10E-04	2.89E-03	1.54E-08
							fucosylation	4.10E-04	4.73E-03	1.24E-05
							bisecting GlcNAc	4.10E-04	2.47E-03	3.04E-06

Supplementary Table 4: Coefficients estimated using the Orkney population for constructing the phenotype scores for replicated new loci.

N-glycosylation

Locus	Marker	coef.GP1	coef.GP2	coef.GP4	coef.GP5	coef.GP6	coef.GP7	coef.GP8	coef.GP9	coef.GP10	coef.GP11	coef.GP12	coef.GP13	coef.GP14	coef.GP15	coef.GP16	coef.GP17	coef.GP18	coef.GP19	coef.GP20	coef.GP21	coef.GP22	coef.GP23	coef.GP24
IGH	rs35590487	-0.02731	-0.0006865	-0.0412559	-0.005335	0.03374699	-0.0108103	-0.0627032	0.03778181	-0.0642565	-0.0109666	-0.0457346	0.0247978	0.03934932	-0.0012902	0.09096162	0.03002213	-0.0790602	-0.0551846	-0.0183474	-0.0314761	0.00924047	-0.0517685	0.02775267
AZI1	rs9319617	0.02671021	-0.0350132	0.11049536	0.03314391	0.04532654	0.1159212	0.0987492	-0.0323572	-0.0127583	-0.0422124	-0.0844758	-0.0572334	0.31728249	0.00755637	0.1317658	0.00833203	-0.1900232	0.09199623	0.02825535	0.02388773	-0.0112682	-0.0229235	-0.0256472
FUT6	rs12019136	0.01262004	-0.0012021	-0.04416	0.0519854	-0.0458217	-0.0382416	0.00321899	0.01198305	0.02456197	0.01790817	0.03879296	-0.0913629	-0.0297934	-0.002679	-0.0484833	-0.0130777	0.04914938	0.0005759	-0.0681638	0.04455286	0.03730474	-0.0379101	-0.013566

digalactosylation

Locus	Marker	coef.GP12	coef.GP13	coef.GP14	coef.GP15	coef.GP17	coef.GP18	coef.GP19	coef.GP21	coef.GP22	coef.GP23	coef.GP24
ELL2	rs11135441	-0.0003488	0.03325104	-0.0052914	-0.0108006	0.05589569	-0.054795	0.08581342	0.01544985	-0.0223953	0.14928937	-0.1841971

galactosylation

Locus	Marker	coef.GP7	coef.GP8	coef.GP9	coef.GP10	coef.GP11	coef.GP12	coef.GP13	coef.GP14	coef.GP15	coef.GP16	coef.GP17	coef.GP18	coef.GP19	coef.GP21	coef.GP22	coef.GP23	coef.GP24
HLA	rs116108880	-0.0604107	-0.0197739	0.04179228	-0.0722957	0.0374587	-0.0360245	0.06356142	-0.0684161	-0.039915	-0.0230286	0.03164473	0.15231182	-0.071164	0.00603239	-0.0230409	-0.1458205	0.09274003
IGH	rs35590487	-0.0012513	-0.066433	0.04444331	-0.026522	-0.0097235	-0.0406829	0.00434598	0.06633653	-0.0110187	0.08785399	0.03348312	-0.0696146	-0.05764	-0.039213	0.00443215	-0.0482837	0.03858519
AZI1	rs9319617	0.07306685	0.04985079	-0.0653394	-0.0339145	-0.0157369	-0.074845	-0.0161444	0.23010129	-0.0343425	0.10978084	-0.0059031	-0.2325809	0.08625044	0.03254167	-0.0100272	-0.0424673	-0.0357071

monogalactosylation

Locus	Marker	coef.GP7	coef.GP8	coef.GP9	coef.GP10	coef.GP11	coef.GP16
IGH	rs35590487	-0.0072524	-0.0595029	0.07811507	0.00507777	-0.0190026	0.04504516

monosialylation

Locus	Marker	coef.GP16	coef.GP17	coef.GP18	coef.GP19
IGH	rs35590487	0.10575164	-0.0077961	-0.0454943	-0.03926

sialylation

Locus	Marker	coef.GP16	coef.GP17	coef.GP18	coef.GP19	coef.GP21	coef.GP22	coef.GP23	coef.GP24
ELL2	rs11135441	-0.0029012	0.05772004	-0.0522041	0.08526415	0.02442217	-0.0136254	0.14324888	-0.1953372
IGH	rs35590487	0.12322356	0.01299612	-0.0430263	-0.016836	-0.0306809	-0.0101108	-0.0010499	-0.025077

fucosylation

Locus	Marker	coef.GP1	coef.GP4	coef.GP6	coef.GP8	coef.GP9	coef.GP10	coef.GP11	coef.GP14	coef.GP15	coef.GP16	coef.GP18	coef.GP19	coef.GP23	coef.GP24
IGH	rs58087925	-0.0362617	0.02198728	0.04014911	-0.0527964	0.04413357	-0.0454584	-0.0301711	0.05159823	0.00156386	0.09391815	-0.0424643	-0.0337934	-0.0597559	0.02116292
AZI1	rs2659009	-0.0502279	-0.0333692	-0.0414107	-0.0952293	0.03540396	0.02541944	-0.001212	-0.2221192	0.02063569	-0.1016911	0.18974476	-0.0675022	-0.0103439	0.02633008

bisecting GlcNAc

Locus	Marker	coef.GP6	coef.GP10	coef.GP11	coef.GP13	coef.GP15	coef.GP19	coef.GP24
IGH	rs8013055	0.00297712	-0.1218838	0.11730341	-0.0382115	0.0384148	-0.0791242	0.02141277

Supplementary Table 5: Database association records showing pleiotropic effects with the novel IgG glycosylation loci (FDR < 5%).

SNP	Position (hg19)	Alleles	Trait	Study	PMID	Year of Publication	Ancestry	P	FDR	N
rs909674	chr22:39859169	A/C	Height	GIANT	25282103	2014	European	4.90E-08	5.32E-07	251250
rs114479369	chr6:31315033	A/G	Schizophrenia	PGC	25056061	2014	Mixed	1.47E-06	1.34E-05	82315
rs4074453	chr14:105998544	T/C	Rheumatoid arthritis	Gregersen PK	19503088	2009	European	2.77E-06	2.41E-05	6922
rs4074453	chr14:105998544	T/C	Mitral Annular Calcification	Thanassoulis G	23388002	2013	Mixed	2.78E-05	2.14E-04	3795
rs4074453	chr14:105998544	T/C	Mitral annular calcium	Thanassoulis G	23388002	2013	Mixed	2.78E-05	2.13E-04	6942
rs8013055	chr14:105990620	A/T	Mitral Annular Calcification	Thanassoulis G	23388002	2013	Mixed	3.33E-05	2.54E-04	3795
rs8013055	chr14:105990620	A/T	Mitral annular calcium	Thanassoulis G	23388002	2013	Mixed	3.33E-05	2.53E-04	6942
rs4074453	chr14:105998544	T/C	Partial epilepsy	Kasperaviciute D	20522523	2010	European	8.48E-05	6.36E-04	10380
rs6421315	chr7:50355207	C/G	Rheumatoid arthritis	Okada Y	24390342	2014	Mixed	0.00022	1.64E-03	80799
rs28733392	chr14:105993258	A/G	Focal Epilepsy	Anney RJ	25087078	2014	Mixed	0.0004081	3.04E-03	28916
rs909674	chr22:39859169	A/C	Schizophrenia	PGC	25056061	2014	Mixed	0.001937	1.42E-02	82315
rs1048775	chr17:79202329	G/C	BMI in males less than or equal to 50 years of age	GIANT	26426971	2015	European	0.0026	1.89E-02	37390
rs8013055	chr14:105990620	A/T	Epilepsy	Anney RJ	25087078	2014	Mixed	0.002968	2.14E-02	34853
rs909674	chr22:39859169	A/C	BMI in females greater than 50 years of age	GIANT	26426971	2015	European	0.0032	2.30E-02	84671
rs909674	chr22:39859169	A/C	Height	GIANT	23754948	2013	European	0.003217	2.30E-02	131808.7
rs6421315	chr7:50355207	C/G	Rheumatoid arthritis	Okada Y	24390342	2014	European	0.0034	2.41E-02	58284
rs12190015	chr6:143182742	A/G	SBP	ICBP	21909115	2011	European	0.00346	2.44E-02	69395
rs12190015	chr6:143182742	A/G	Systolic blood pressure SBP	ICBP	21909115	2011	European	0.00346	2.43E-02	69395
rs11847263	chr14:65775695	G/T	Acute lung injury following major trauma	Christie	22295056	2012	European	0.00393	2.75E-02	2866
rs58087925	chr14:105983096	C/T	CHD	CARDIoGRAMplusC4D	26343387	2015	Mixed	0.004071	2.84E-02	184305
rs35590487	chr14:105989599	C/T	Rheumatoid arthritis	Okada Y	24390342	2014	Mixed	0.0041	2.84E-02	80799
rs8013055	chr14:105990620	A/T	Focal Epilepsy	Anney RJ	25087078	2014	Mixed	0.004148	2.87E-02	28916
rs35590487	chr14:105989599	C/T	Rheumatoid arthritis	Okada Y	24390342	2014	European	0.0042	2.89E-02	58284
rs11135441	chr5:95222277	C/T	Crohns disease	IBDGC	23128233	2012	European	0.0043	2.95E-02	14342
rs8013055	chr14:105990620	A/T	Rheumatoid arthritis	Okada Y	24390342	2014	European	0.0044	3.00E-02	58284
rs8013055	chr14:105990620	A/T	Rheumatoid arthritis	Okada Y	24390342	2014	Mixed	0.0044	2.99E-02	80799
rs2186369	chr22:24170996	G/T	Waist hip ratio adjusted for BMI in males greater than 50 years of age	GIANT	26426971	2015	European	0.0045	3.05E-02	39460
rs12190015	chr6:143182742	A/G	Fasting blood glucose	MAGIC	20081858	2010	European	0.00459	3.09E-02	46186
rs12190015	chr6:143182742	A/G	Fasting glucose	MAGIC	20081858	2010	European	0.00459	3.10E-02	46186
rs58087925	chr14:105983096	C/T	Epilepsy	Anney RJ	25087078	2014	Mixed	0.004616	3.09E-02	34853
rs28733392	chr14:105993258	A/G	CHD	CARDIoGRAMplusC4D	26343387	2015	Mixed	0.004859	3.24E-02	184305
rs4074453	chr14:105998544	T/C	Rheumatoid arthritis	Okada Y	24390342	2014	European	0.0049	3.26E-02	58284
rs909674	chr22:39859169	A/C	BMI	GIANT	25673413	2015	Mixed	0.005341	3.54E-02	236053
rs28733392	chr14:105993258	A/G	Rheumatoid arthritis	Okada Y	24390342	2014	European	0.0056	3.69E-02	58284
rs58087925	chr14:105983096	C/T	Rheumatoid arthritis	Okada Y	24390342	2014	Mixed	0.0058	3.81E-02	80799
rs58087925	chr14:105983096	C/T	Rheumatoid arthritis	Okada Y	24390342	2014	European	0.0058	3.80E-02	58284
rs8013055	chr14:105990620	A/T	CHD	CARDIoGRAMplusC4D	26343387	2015	Mixed	0.006017	3.92E-02	184305
rs58087925	chr14:105983096	C/T	Focal Epilepsy	Anney RJ	25087078	2014	Mixed	0.006643	4.32E-02	28916
rs12342831	chr9:33124872	C/T	Genetic Generalised Epilepsy	Anney RJ	25087078	2014	Mixed	0.006828	4.42E-02	21596
rs11135441	chr5:95222277	C/T	BMI in females less than or equal to 50 years of age	GIANT	26426971	2015	European	0.0069	4.45E-02	54258
rs2186369	chr22:24170996	G/T	Birth weight	EGGC	23202124	2013	European	0.0072	4.61E-02	26836
rs35590487	chr14:105989599	C/T	CHD	CARDIoGRAMplusC4D	26343387	2015	Mixed	0.007337	4.66E-02	184305
rs6421315	chr7:50355207	C/G	Fasting blood glucose	MAGIC	20081858	2010	European	0.007571	4.78E-02	46186
rs6421315	chr7:50355207	C/G	Fasting glucose	MAGIC	20081858	2010	European	0.007571	4.79E-02	46186

Supplementary Table 6: Extended results of the novel loci detected via multivariate GWAS for IgG *N*-glycosylation. Nine multivariate GWA scans were performed, including one using all the 23 phenotypes, as well as eight different subgroupings according to type of glycosylation. Replication was performed by (i) MANOVA test in the replication cohorts (P_{MANOVA}); and (ii) testing the association between the phenotypic score (constructed based on the coefficients estimated in the discovery cohort) and the corresponding genotype dosages (P_S, reported only for replication cohorts). β_S denotes the coefficient of regression of genotype dosage onto the phenotypic score. Consistency of effects was performed by testing the correlation of partial genotype-phenotype correlations (r_ρ) in the discovery and replication cohorts. *f*: frequency; R^2: imputation R-squared.

Chr	Candidate Genes	Phenotypes Grouping	Number of Phenotypes	Top Variant	A1	A0	Discovery: ORCADES				Replication I: KORCULA + VIS						Replication II: TWINSUK						Combined	
							f(A1)	R^2	β_S (s.e.)	*P*	*f*(A1)	R^2	P_{MANOVA}	β_S (s.e.)	P_S	r_ρ (95% C.I.)	*f*(A1)	R^2	P_{MANOVA}	β_S (s.e.)	P_S	r_ρ (95% C.I.)	β_S (s.e.)	$P_{S,\text{meta}}$
5	*ELL2*	digalactosylation	11	rs11135441	T	C	0.32	0.99	0.0336 (0.0056)	1.6E-09	0.30	0.96	4.5E-04	0.0276 (0.0058)	2.1E-06	0.60 (0.08, 0.83)	0.30	0.96	6.9E-09	0.0183 (0.0027)	5.5E-12	0.86 (0.53, 0.93)	0.0221 (0.0022)	5.4E-23
		sialylation	8	rs11135441	T	C	0.32	0.99	0.0313 (0.0050)	5.0E-10	0.30	0.96	1.1E-04	0.0234 (0.0056)	2.7E-05	0.76 (0.07, 0.91)	0.30	0.96	3.6E-09	0.0091 (0.0014)	2.7E-10	0.96 (0.75, 0.98)	0.0114 (0.0013)	3.2E-18
6	*HLA-B-C*	galactosylation	17	rs116108880	G	C	0.60	0.96	0.0408 (0.0066)	8.3E-10	0.72	0.99	6.2E-01	0.0188 (0.0053)	4.0E-04	0.39 (-0.42, 0.76)	0.67	0.98	3.8E-08	0.0160 (0.0031)	2.2E-07	0.74 (0.14, 0.86)	0.0201 (0.0025)	5.0E-16
14	*IGH*	*N*-glycosylation	23	rs35590487	T	C	0.23	0.99	0.0671 (0.0080)	6.3E-17	0.29	1.00	2.3E-02	0.0218 (0.0074)	3.1E-03	0.10 (-0.38, 0.54)	0.24	1.00	1.0E-31	0.0534 (0.0040)	5.2E-41	0.76 (0.16, 0.83)	0.0496 (0.0032)	1.4E-53
		monogalactosylation	6	rs35590487	T	C	0.23	0.99	0.0465 (0.0055)	3.6E-17	0.29	1.00	7.1E-02	0.0178 (0.0040)	9.5E-06	0.90 (0.09, 0.96)	0.24	1.00	6.3E-25	0.0358 (0.0032)	1.2E-28	0.86 (0.34, 0.97)	0.0318 (0.0023)	2.0E-44
		galactosylation	17	rs35590487	T	C	0.23	0.99	0.0637 (0.0073)	3.4E-18	0.29	1.00	8.8E-02	0.0212 (0.0059)	3.3E-04	0.02 (-0.49, 0.65)	0.24	1.00	4.7E-30	0.0501 (0.0039)	4.1E-38	0.85 (0.29, 0.90)	0.0450 (0.0030)	7.5E-52
		monosialylation	4	rs35590487	T	C	0.23	0.99	0.0325 (0.0046)	9.9E-13	0.29	1.00	3.5E-01	0.0114 (0.0044)	9.9E-03	0.99 (-0.45, 0.99)	0.24	1.00	6.3E-24	0.0287 (0.0026)	1.2E-27	0.97 (0.84, 1.00)	0.0258 (0.0020)	1.2E-37
		sialylation	8	rs35590487	T	C	0.23	0.99	0.0379 (0.0054)	1.5E-12	0.29	1.00	3.5E-01	0.0113 (0.0050)	2.3E-02	0.36 (-0.37, 0.76)	0.24	1.00	5.9E-23	0.0287 (0.0026)	1.2E-27	0.94 (0.60, 0.96)	0.0270 (0.0021)	4.5E-37
		fucosylation	14	rs58087925	T	C	0.22	0.91	0.0625 (0.0070)	3.8E-19	0.29	1.00	7.2E-02	0.0242 (0.0059)	4.5E-05	0.18 (-0.38, 0.65)	0.24	1.00	3.7E-34	0.0523 (0.0038)	8.3E-43	0.93 (0.43, 0.94)	0.0472 (0.0029)	2.1E-59
		bisecting GlcNAc	7	rs8013055	A	T	0.22	0.94	0.0370 (0.0052)	1.0E-12	0.29	1.00	3.0E-01	0.0077 (0.0058)	1.8E-01	0.70 (-0.16, 0.87)	0.24	1.00	1.1E-15	0.0261 (0.0029)	1.9E-19	0.96 (0.78, 0.99)	0.0253 (0.0023)	1.0E-27
17	*AZI1*	*N*-glycosylation	23	rs9319617	C	T	0.52	0.98	0.0453 (0.0076)	2.5E-09	0.45	0.97	9.7E-03	0.0204 (0.0064)	1.4E-03	0.69 (0.06, 0.79)	0.49	0.96	7.6E-07	0.0199 (0.0033)	2.2E-09	0.75 (0.03, 0.85)	0.0232 (0.0027)	1.8E-17
		galactosylation	17	rs9319617	C	T	0.52	0.98	0.0422 (0.0073)	2.7E-09	0.45	0.97	2.6E-03	0.0189 (0.0047)	6.2E-05	0.79 (0.16, 0.87)	0.49	0.97	1.2E-06	0.0176 (0.0029)	8.5E-10	0.78 (-0.11, 0.89)	0.0204 (0.0023)	2.2E-18
		fucosylation	14	rs2659009	A	G	0.50	0.91	0.0414 (0.0063)	3.9E-11	0.50	0.98	5.1E-04	0.0168 (0.0049)	5.8E-04	0.85 (0.13, 0.91)	0.44	0.93	6.0E-05	0.0159 (0.0027)	4.0E-09	0.89 (-0.04, 0.92)	0.0192 (0.0022)	3.7E-16
19	*FUT6-3*	*N*-glycosylation	23	rs12019136	A	G	0.05	0.93	0.0574 (0.0078)	1.9E-13	0.41	0.96	3.3E-01	0.0022 (0.0070)	7.5E-01	0.01 (-0.41, 0.39)	0.42	1.00	3.3E-15	0.0251 (0.0031)	6.3E-16	0.82 (0.31, 0.82)	0.0256 (0.0027)	8.7E-22
